# Beyond Protein Restriction: Comparative eADI-17-Estimated Anti-Inflammatory Profiles of Four Renal Diets

**DOI:** 10.3390/nu18203395

**Published:** 2026-10-16

**Authors:** Claudia D’Alessandro, Adamasco Cupisti, Vincenzo Panichi, Diego Moriconi, Domenico Giannese

**Affiliations:** Department of Clinical and Experimental Medicine, University of Pisa, 56126 Pisa, Italy; claudia.dalessandro@unipi.it (C.D.);

**Keywords:** chronic kidney disease, renal diet, inflammation, empirical Anti-inflammatory Diet Index, eADI-17, low-protein diet, Mediterranean renal diet

## Abstract

Background/Objectives: Chronic low-grade inflammation contributes to chronic kidney disease (CKD) progression and cardiovascular complications. Although nutritional therapy is central to CKD management, the estimated anti-inflammatory profiles of renal diets remain poorly characterized. Methods: We analyzed 40 daily menus with broadly comparable, although not identical, energy content (10 per dietary pattern): Mediterranean renal diet (MedRen; 0.8 g protein/kg), vegan diet (0.7 g/kg), conventional low-protein diet (LPD; 0.6 g/kg), and supplemented very-low-protein diet (sVLPD; 0.3 g/kg). Dietary anti-inflammatory potential was estimated using the 17-component empirical Anti-inflammatory Diet Index (eADI-17), with higher scores indicating a more favorable profile. Groups were compared using exploratory non-parametric tests with multiple-comparison correction; nutrient associations were assessed using Spearman correlations with false-discovery-rate correction. Results: Mean eADI-17 scores were 11.90 ± 0.66 for MedRen, 9.75 ± 0.63 for vegan, 8.90 ± 1.10 for LPD, and 8.40 ± 0.52 for sVLPD. MedRen scored higher than all other diets, and vegan scored higher than sVLPD. In pooled analyses, eADI-17 correlated positively with total protein and phosphorus and negatively with vitamin C and total carbohydrate; none of these associations remained significant after accounting for dietary-group membership. Conclusions: The eADI-17-estimated anti-inflammatory potential of renal diets depends on the overall dietary pattern and does not necessarily improve with greater protein restriction. Because eADI-17 is an indirect food-based index and inflammatory biomarkers were not measured, these findings are hypothesis-generating and require validation in studies of actual intake, biomarkers, and clinical outcomes.

## 1. Introduction

Chronic low-grade inflammation accompanies the development of many chronic diseases, including chronic kidney disease (CKD) [1,2].

In CKD, inflammation contributes to endothelial dysfunction, glomerular hypertension, progressive kidney damage, and cardiovascular complications [3,4]. Among several risk factors, lifestyle factors, including diet, may initiate or sustain this inflammatory state.

Specific nutrients and foods can influence circulating inflammatory biomarkers. Plant-derived foods, whole grains, and legumes are generally associated with a more favorable inflammatory profile, whereas processed meat and added sugars are commonly associated with a less favorable profile [5,6].

Several dietary indices have been developed to estimate the inflammatory potential of diet [6]. The Dietary Inflammatory Index (DII) is a literature-derived, nutrient-based score [7], whereas the Empirical Dietary Inflammatory Index (EDII) is a food-group-based score derived using reduced-rank regression and stepwise linear regression [8]. More recently, Kaluza et al. developed the 17-component empirical Anti-inflammatory Diet Index (eADI-17), a user-friendly food-based score associated with serum levels of high-sensitivity C-reactive protein, interleukin-6, TNF receptor 1, and TNF receptor 2. The final index includes food groups consistently selected in at least 9 of 10 cross-validation folds; higher scores indicate a more favorable eADI-17-estimated anti-inflammatory profile [9].

Dietary therapy is central to the management of non-dialysis CKD. When appropriately prescribed and implemented, it can help prevent or correct uremic metabolic abnormalities, delay kidney replacement therapy, maintain nutritional status, and reduce cardiovascular risk [10].

Diet is also a modifiable determinant of kidney health and CKD outcomes [11,12].

In 23,175 middle-aged and older NHANES participants, Guo et al. found that higher DII scores were independently associated with CKD and low eGFR, but not with albuminuria after full adjustment [13].

Persistent low-grade inflammation is also associated with adverse kidney outcomes. In adults with atherosclerotic cardiovascular disease, elevated CRP predicted acute kidney injury and faster kidney function decline, supporting inflammation as a potential link between cardiovascular and kidney disease progression [14,15].

Pro-inflammatory cytokines promote muscle protein catabolism and suppress muscle synthesis, providing a mechanistic link between inflammation and sarcopenia [16,17]. In patients with CKD, a higher DII has been independently associated with greater odds of sarcopenia, although the cross-sectional design precludes causal inference [18].

This study aimed to compare the eADI-17-estimated anti-inflammatory potential of four commonly prescribed renal dietary patterns using the eADI-17 and to identify food-group characteristics that may explain between-diet differences.

## 2. Materials and Methods

We calculated the nutrient composition of four renal diets that were primarily distinguished by protein content and had broadly comparable, although not identical, energy content: Mediterranean renal diet (MedRen; 0.8 g protein/kg body weight), vegan diet (0.7 g/kg), conventional low-protein diet (LPD; 0.6 g/kg), and supplemented very-low-protein diet (sVLPD; 0.3 g/kg) [19].

The dietary patterns were designed to provide similar overall energy prescriptions while preserving the nutritional characteristics and food-selection principles specific to each renal diet. Consequently, energy contents were broadly comparable but not identical across menu sets.

For each dietary pattern, 10 daily meal plans for a standardized 70 kg patient were selected from sets routinely used during or after nutritional counseling for patients with CKD, yielding 40 menus in total. The menus were neither randomly sampled from a larger database nor developed specifically for this analysis. They were selected to provide practical day-to-day variety while meeting the protein target and food-selection principles of the corresponding dietary prescription.

Within each dietary group, the menus were alternative daily implementations of the same underlying prescription and therefore were not fully independent biological observations. The menu, rather than the patient, was the unit of analysis. Accordingly, inferential analyses were considered exploratory and descriptive of the selected menu sets.

The main characteristics and prescriptive principles of the four dietary patterns are summarized in Table 1. Nutrient-characterization variables were summarized as daily mean values across the 10 menus in each group.

Energy and nutrient contents were obtained from the CREA Food Composition Tables and the Food Composition Database for Epidemiological Studies in Italy [20,21]. Vitamin K1 content was derived from the United States Department of Agriculture Food Data Central database [22]. Potential renal acid load (PRAL) and net endogenous acid production (NEAP) were also calculated. PRAL was estimated according to Remer and Manz: PRAL (mEq/day) = 0.49 × protein (g/day) + 0.037 × phosphorus (mg/day) − 0.021 × potassium (mg/day) − 0.026 × magnesium (mg/day) − 0.013 × calcium (mg/day). NEAP was estimated according to Frassetto et al.: NEAP (mEq/day) = 54.5 × protein (g/day)/potassium (mmol/day) − 10.2 [23,24].

### 2.1. Estimation of eADI-17 Anti-Inflammatory Potential

The empirical Anti-inflammatory Diet Index (eADI-17) was used to estimate the anti-inflammatory potential of each menu [9]. It was selected because it is a recently developed empirical food-based index associated with several circulating inflammatory biomarkers and designed to characterize whole dietary patterns rather than isolated nutrients. A food-based approach was therefore aligned with our objective of comparing complete renal dietary prescriptions. However, eADI-17 was developed in older Swedish men and has not been validated in patients with CKD or in Italian dietary settings; its use here is an exploratory external application.

**Figure 1:**
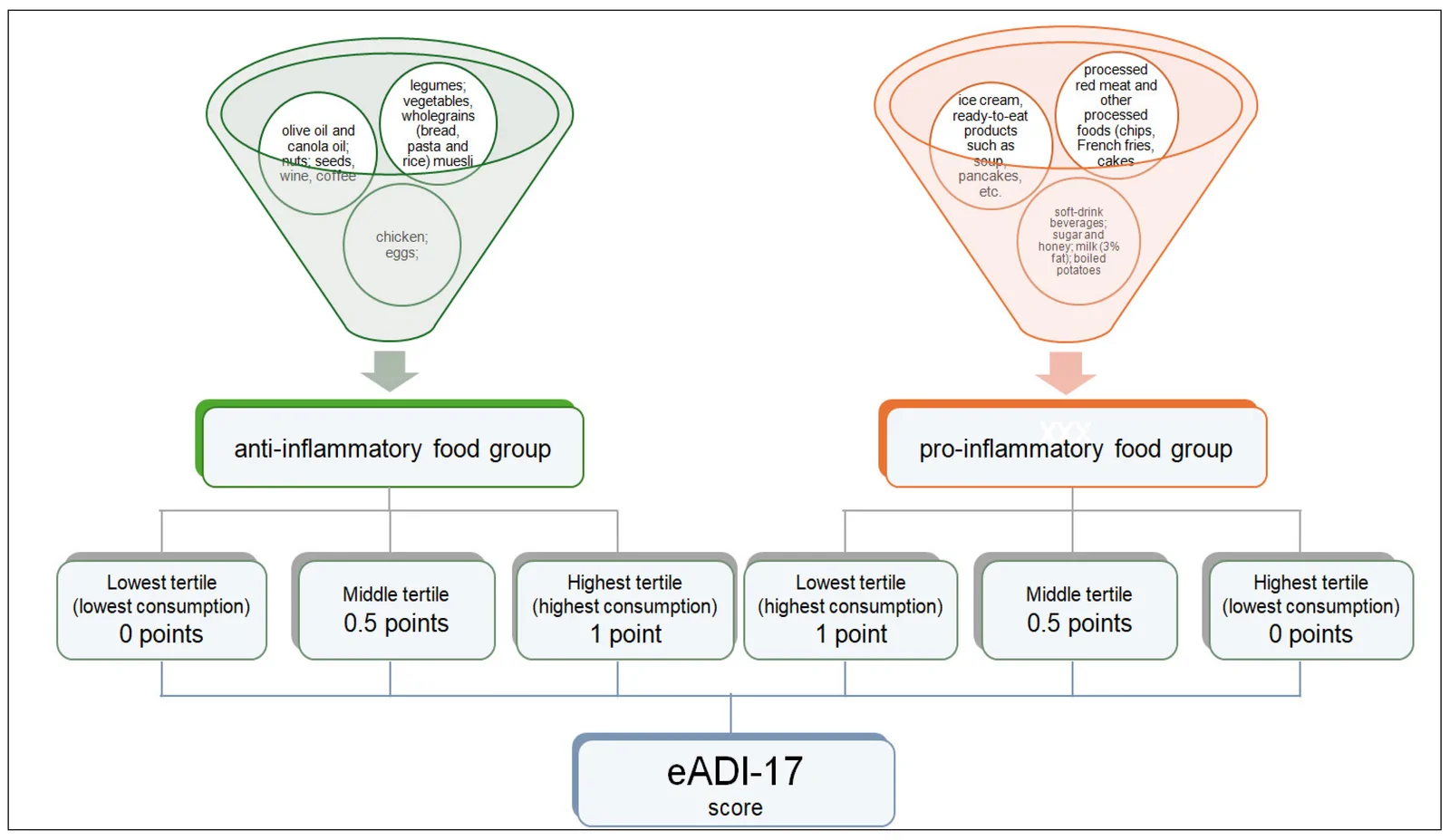
Calculation of the empirical Anti-inflammatory Diet Index-17 (eADI-17). The consumption cut-offs developed by Kaluza et al. in older Swedish men were applied after assigning foods in the Italian renal menus to the corresponding eADI-17 food groups. This is an exploratory external application; the cut-offs are not validated clinical thresholds for patients with CKD.

The index includes 17 food groups: 11 classified as anti-inflammatory and six as pro-inflammatory (Figure 1). Menu foods and beverages were assigned to these components using the definitions and consumption cut-offs reported by Kaluza et al. [9]. Mixed dishes and composite foods were disaggregated into their component ingredients; only ingredients matching an eADI-17 food-group definition contributed to the score. Protein-free products were not included in the eADI-17 calculation because they are not represented among the food-group components of the original index. KA/EAA supplements contributed to the reported nutrient composition when applicable, but they are likewise not represented in the eADI-17 food-group structure and therefore did not contribute to the score. The operational mapping and original scoring thresholds are summarized in Table 2.

For anti-inflammatory components, the lowest, middle, and highest intake categories receive 0, 0.5, and 1 point, respectively; scoring is reversed for pro-inflammatory components. Component scores are summed to obtain the total eADI-17 score, with higher values indicating a dietary pattern more closely aligned with the favorable food-group profile captured by the index (Table 2).

### 2.2. Statistical Analysis

Quantitative variables were summarized as mean and standard deviation; eADI-17 scores were also described using medians and interquartile ranges. Because menus within each group were alternative implementations of a common prescription rather than independent biological observations, all inferential analyses were considered exploratory. Descriptive measures of central tendency, dispersion, and absolute between-group differences were treated as the principal results; hypothesis tests were used only as complementary summaries of separation among the selected menu sets.

Associations between eADI-17 and energy, macro- and micronutrient contents were assessed across all 40 menus using Spearman’s rank correlation coefficient (ρ). Correlation *p*-values were adjusted with the Benjamini–Hochberg procedure to control the false discovery rate (FDR). q-values < 0.05 were considered statistically significant.

Differences in the eADI-17 distribution among the four dietary groups were assessed using the Kruskal–Wallis test.

When the overall test was statistically significant, two-sided Mann–Whitney U tests were performed for pairwise post hoc comparisons. The *p*-values from the six pairwise comparisons were adjusted using Holm’s sequential procedure to control the family-wise type I error rate. Holm-adjusted *p*-values < 0.05 were considered statistically significant.

To determine whether pooled nutrient-eADI-17 associations persisted beyond between-group compositional differences, we calculated partial Spearman correlations. eADI-17 and each nutrient variable were rank-transformed, and dietary group was entered as a categorical covariate using three indicator variables. The partial correlation was obtained from the association between residualized ranks after removal of the variance attributable to dietary-group membership. Two-sided *p*-values were calculated for each partial correlation and adjusted across nutrient tests using the Benjamini–Hochberg false discovery rate; q-values < 0.05 were considered statistically significant. Because menus shared a common prescriptive framework within each group, these analyses remain exploratory.

## 3. Results

The analysis included 40 menus, equally distributed among the MedRen (0.8 g protein/kg), vegan (0.7 g/kg), conventional LPD (0.6 g/kg), and sVLPD (0.3 g/kg) groups. General characteristics are shown in Table 1, the operational eADI-17 mapping in Table 2, mean energy and nutrient contents, PRAL, and NEAP in Table 3, and eADI-17 scores in Table 4.

The distributions of eADI-17 scores differed descriptively among dietary patterns. Exploratory Kruskal–Wallis testing was consistent with this separation (Table 4). In pairwise Mann–Whitney U tests with Holm correction, MedRen had higher eADI-17 values than each of the other diets, and vegan had higher values than sVLPD (Table 5). Differences between LPD and vegan and between LPD and sVLPD were not statistically significant; these results should not be interpreted as evidence of equivalence.

A non-significant result does not demonstrate equivalence between groups. With only 10 menus per group, the analysis may have had limited power to detect smaller differences.

Across all 40 menus, eight nutritional variables were correlated with eADI-17 after FDR correction (Table 6).

Higher eADI-17 values were most strongly associated with greater total protein and phosphorus contents. Moderate positive associations were observed for plant protein, zinc, iron, and calcium. Conversely, higher vitamin C and total carbohydrate contents were associated with lower eADI-17 values. These correlations should not be interpreted as evidence that individual nutrients independently improve or worsen the eADI-17-anti-inflammatory potential of the diets. Because the analyses pooled menus from different dietary patterns, the observed associations may largely reflect compositional differences among the dietary groups rather than direct nutrient-specific relationships.

Fiber, energy, lipids, sodium, copper, vitamin B1, and water had nominal *p*-values below 0.05 but did not remain significant after FDR correction. These findings should therefore not be regarded as statistically conclusive.

**Figure 2:**
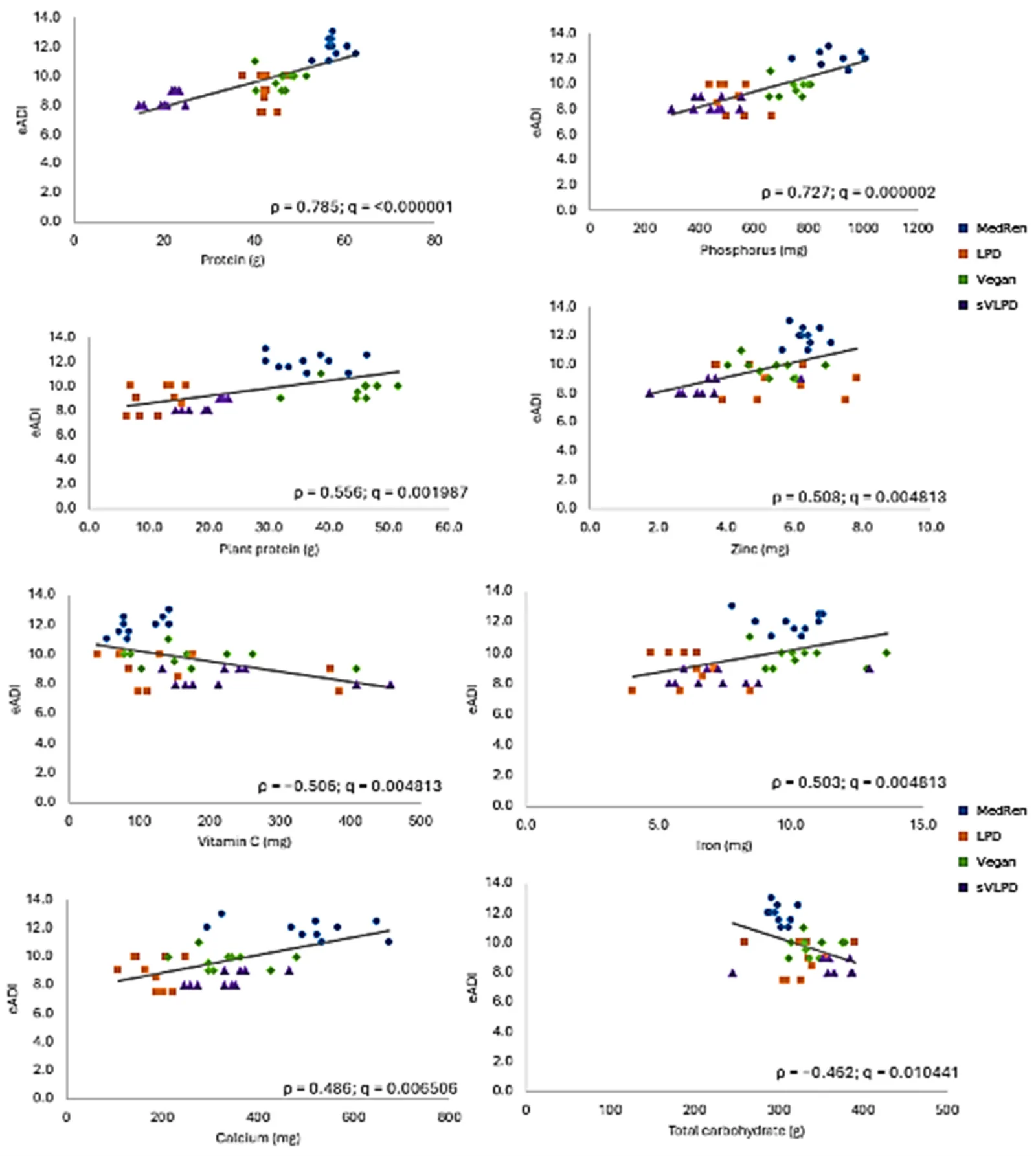
Pooled menu-level associations between eADI-17 and selected nutritional characteristics of the renal diets. Only correlations that remained significant after FDR correction in the unadjusted pooled analysis are shown. These associations may reflect structural differences among dietary patterns and should not be interpreted as independent nutrient effects.

The pooled correlations may reflect compositional differences among dietary groups (Figure 2). After dietary-group membership was accounted for using partial Spearman correlations, none of the nutrient-eADI-17 associations remained significant following FDR correction.

This result suggests that the pooled associations primarily reflected structural differences among the four dietary patterns rather than independent nutrient-specific effects. The positive correlations for protein and phosphorus and the negative correlation for vitamin C therefore should not be interpreted causally or as evidence that these nutrients directly improve or worsen the eADI-17-estimated anti-inflammatory profile.

## 4. Discussion

This menu-based analysis found that eADI-17 scores differed across the selected renal dietary patterns. MedRen had the highest score, followed by the vegan diet, conventional LPD, and sVLPD. At the descriptive level, there was limited overlap between the highest-scoring MedRen menus and the lowest-scoring sVLPD menus. The magnitude and consistency of these observed differences are more informative for this menu-based comparison than statistical significance alone. Because eADI-17 scoring is based on absolute food-group intake thresholds, the lower mean energy content of the MedRen menus compared with the other dietary groups may have influenced food-group quantities and contributed, at least in part, to the observed between-group differences. The direction and magnitude of this possible influence cannot be determined from the present analysis, and the comparisons should therefore be interpreted cautiously.

Kaluza et al. reported median eADI-17 scores of 8.5 and 9.0 in the discovery and replication cohorts, respectively (range, 2–16 in both cohorts). In the replication cohort, participants with scores ≥ 12.5 had 55.0% lower median hsCRP, 15.2% lower IL-6, 8.2% lower TNF-R1, and 9.5% lower TNF-R2 concentrations than those with scores ≤ 6 [9]. These distribution-based categories are not validated clinical cut-offs. Accordingly, the lower sVLPD score should be interpreted comparatively and does not establish that the diet is intrinsically pro-inflammatory. Rather, the findings suggest that greater protein restriction does not automatically translate into a more favorable eADI-17 profile.

An additional methodological consideration concerns the external application of the eADI-17 to therapeutic renal diets. The index was originally developed in free-living older Swedish adults and was not specifically designed to evaluate dietary prescriptions used in CKD management. Renal diets often include food substitutions, protein-free products, and ketoacid/amino acid supplements introduced to achieve clinical targets. Protein-free products and KA/EAA supplements were not included in the eADI-17 calculation because they are not represented among the original food-group components. Consequently, differences in eADI-17 scores may partly reflect the compatibility of a renal dietary pattern with the food groups incorporated in the index rather than actual differences in biological inflammatory activity. Clinical studies incorporating inflammatory biomarkers are required to determine whether these score differences correspond to measurable differences in inflammatory status.

Although several eADI-17 components overlap with foods commonly emphasized in Mediterranean and DASH dietary patterns, direct comparisons should be interpreted cautiously because validated eADI-17 estimates for these dietary models are not currently available.

From a practical perspective, evaluating the balance of eADI-17 food groups may help clinicians identify potential improvements without introducing unnecessary restrictions. Such changes must preserve adequate energy intake, prescribed protein targets, mineral and acid–base control, nutritional status, dietary habits, and acceptability. The eADI-17 should therefore not be maximized in isolation but integrated with the established therapeutic priorities of each renal diet and the individual patient’s clinical and nutritional needs.

Strengths of this study include the standardized comparison of four clinically used dietary patterns, the analysis of multiple daily menus rather than a single representative menu, and the use of an empirically developed food-group-based index. Correction for multiple comparisons and an exploratory analysis accounting for dietary-group membership reduced the risk of overinterpreting nutrient-level associations. Given the limited number of menus per dietary pattern and their shared underlying prescription framework, the statistical results should be interpreted primarily as descriptive indicators of differences among the selected menu collections. Because the analysis was based on structured menu sets rather than independent patient observations, emphasis should be placed on the descriptive separation between dietary patterns and on the consistency of the observed rankings across menus. In this context, statistical significance should be viewed as supportive rather than definitive evidence.

The observed differences represent eADI-17-estimated anti-inflammatory potential rather than demonstrated biological effects; the findings should therefore be considered hypothesis-generating.

## 5. Limitations

Several limitations should be acknowledged. First, the analysis was based on prescribed menus for a standardized 70 kg patient rather than observed intake; adherence, substitutions, portion-size variation, and food preparation were not captured. Second, the 10 menus within each group were alternative implementations of the same prescriptive framework and were not independent biological observations. The small number of menus per pattern and their shared origin limit conventional inference; *p*-values should therefore be regarded as exploratory descriptors of separation among the selected menu sets, not as population-level evidence. Third, energy intake was broadly comparable but not identical across groups, which may have influenced food-group quantities and eADI-17 scores. Fourth, eADI-17 is an indirect food-based estimate, and inflammatory biomarkers were not measured. Fifth, the index and its cut-offs were developed in older Swedish men and have not been validated in patients with CKD or in Italian dietary settings. Its transferability to therapeutic renal diets is uncertain, particularly because protein-free products and KA/EAA supplements were excluded from the eADI-17 calculation. Finally, correlated differences in protein quantity, protein source, food groups, and micronutrients prevent attribution of the observed scores to individual dietary components.

Importantly, the present findings should not be interpreted as evidence that any evaluated dietary pattern reduces or increases inflammation in patients with CKD. The eADI-17 estimates how closely menu composition aligns with a food-group profile associated with inflammatory biomarkers; it is not a direct measure of systemic inflammation. Because C-reactive protein, interleukin-6, and tumor necrosis factor-related markers were not measured, score differences may reflect compatibility with the eADI-17 framework rather than genuine differences in inflammatory biology. These findings are hypothesis-generating and require confirmation in studies combining observed dietary intake with inflammatory biomarkers and patient-centered outcomes.

Future studies should evaluate eADI-17 in freely consumed renal diets and test its association with inflammatory biomarkers and clinically relevant outcomes. Prospective interventions should also determine whether targeted changes in food-group composition can improve the estimated inflammatory profile without compromising adherence, nutritional status, kidney function, cardiovascular risk, muscle health, or quality of life.

## 6. Conclusions

In this exploratory menu-based analysis, four renal diets differed in their eADI-17-estimated anti-inflammatory profiles. MedRen had the highest score and sVLPD the lowest; greater protein restriction therefore did not necessarily correspond to a more favorable estimated profile.

Associations between eADI-17 and individual nutrients largely reflected structural differences among dietary groups and should not be interpreted causally. In particular, the pooled positive associations with total protein and phosphorus and the negative association with vitamin C do not support increasing protein or phosphorus intake or reducing vitamin C intake.

The findings describe menu-based estimates rather than demonstrated inflammatory effects. Studies of freely consumed renal diets that include inflammatory biomarkers and clinically relevant outcomes are needed before clinical implications or protective effects can be established.

## Figures and Tables

**Table 1:** General characteristics of the four renal dietary patterns whose eADI-17-estimated anti-inflammatory potential was assessed.

Feature	MedRen	LPD	Vegan	sVLPD
**Protein target**	0.8 g/kg	0.6 g/kg	0.7 g/kg	0.3 g/kg
**Animal foods**	Moderate	Daily HBV protein	Excluded	Excluded
**Legumes**	≥3/week	1–2/week	Daily	Limited
**Cereals**	Daily	Limited	Daily	Limited
**Vegetables & fruit**	Daily	Daily	Daily	Daily
**Dairy**	Limited	Excluded	Excluded	Excluded
**Protein-free foods**	No	Yes	No	Yes
**KA/EAA**	No	Optional	Optional	Mandatory

Note: In clinical practice, food choices may vary according to comorbidities and individual nutritional needs. Fruit and vegetable prescriptions were adjusted to potassium requirements. KA/EAA, keto acid analogues and essential amino acids.

**Table 2:** Operational mapping and original scoring thresholds for the 17 eADI-17 components.

Component	Direction	Operational Handling in Renal Menus	Original Categories for 0/0.5/1 Point
**Whole grains**	Anti-inflammatory	Whole-grain bread, pasta, rice, and other qualifying whole grains	<2.5/2.5–4.0/>4.0 servings/day
**Vegetables**	Anti-inflammatory	Qualifying vegetables, including ingredients of mixed dishes	<2.2/2.2–3.8/>3.8 servings/day
**Coffee**	Anti-inflammatory	Coffee as consumed in the menu	<2.0/2.0–3.0/>3.0 servings/day
**Olive oil**	Anti-inflammatory	Use for cooking or dressing; original use-point system retained	0/1/>1 servings/day
**Chicken**	Anti-inflammatory	Chicken portions, including identifiable mixed-dish ingredients	0/0.1–0.5/>0.5 servings/week
**Eggs**	Anti-inflammatory	Eggs and identifiable egg portions	<1.0/1.0–2.0/>2.0 servings/week
**Legumes**	Anti-inflammatory	Beans, lentils, chickpeas, and other legumes	0/0.1–1.0/>1.0 servings/week
**Muesli**	Anti-inflammatory	Muesli when specifically present	0/0.1–3.5/>3.5 servings/week
**Nuts**	Anti-inflammatory	Nuts when specifically present	0/0.1–1.0/>1.0 servings/week
**Seeds**	Anti-inflammatory	Seeds when specifically present	0/0.1–1.0/>1.0 servings/week
**Wine**	Anti-inflammatory	Wine when included in the prescribed menu	<0.2/0.2–1.5/>1.5 servings/week
**Processed red meat**	Pro-inflammatory	Ham, salami, and other processed red meat	>1.5/0.8–1.5/<0.8 servings/day
**processed foods**	Pro-inflammatory	Qualifying processed foods; composite products disaggregated when possible	>2.5/1.5–2.5/<1.5 servings/day
**Boiled potatoes**	Pro-inflammatory	Boiled potato portions	>0.7/0.5–0.7/<0.5 servings/day
**Milk (3% fat)**	Pro-inflammatory	Milk matching the original component definition	>0.9/0.1–0.9/0 servings/day
**Sugar and honey**	Pro-inflammatory	Added sugar and honey; qualifying portions in composite foods	>0.9/0.1–0.9/0 servings/day
**Soft drinks**	Pro-inflammatory	Sugar-sweetened soft drinks	>0.2/0.1–0.2/0 servings/day

**Table 3:** Mean energy and nutrient content of the four renal diets.

Parameter	MedRen	LPD	Vegan Diet	sVLPD
Energy (kcal/day)	2012 ± 54	2229 ± 98	2190 ± 88	2187 ± 201
Protein (g/day)	57.6 ± 2.6	42.5 ± 2.6	45.9 ± 3.6	20.4 ± 3.3
Animal protein (g/day)	17.2 ± 7.8	24.9 ± 10.2	0.9 ± 1.6	0.6 ± 1.1
Plant protein (g/day)	36.5 ± 5.7	11.3 ± 3.7	44.6 ± 5.5	19.5 ± 3.1
Total fat (g/day)	72.2 ± 5.9	91.3 ± 14.5	79.3 ± 9.8	82.9 ± 14.3
Saturated fat (g/day)	15.1 ± 3.9	18.9 ± 5.8	17.4 ± 6.5	17.2 ± 4.8
Unsaturated fat (g/day)	49.9 ± 3.1	57.3 ± 7.9	55.4 ± 8.7	50.8 ± 11.5
Carbohydrates (g/day)	302 ± 11.9	328 ± 33.7	341 ± 23.0	358 ± 42.0
Starches (g/day)	196 ± 11.2	174 ± 34.9	235 ± 17.8	179 ± 54.3
Sugars (g/day)	96.3 ± 8.9	86.9 ± 18.0	96.0 ± 10.6	105 ± 11.0
Fiber (g/day)	29.7 ± 4.6	17.8 ± 3.1	34.7 ± 4.2	26.1 ± 3.5
Cholesterol (mg/day)	108 ± 90.6	107 ± 23.0	33.7 ± 39.2	39.7 ± 32.7
Calcium (mg/day)	505 ± 122	182 ± 42.0	334 ± 76.3	334 ± 64.8
Phosphorus (mg/day)	899 ± 83	527 ± 66	745 ± 56	444 ± 79
Sodium (mg/day)	620 ± 189	373 ± 224	647 ± 653	352 ± 207
Potassium (mg/day)	2314 ± 426	2007 ± 280	3152 ± 685	2753 ± 575
Iron (mg/day)	10.0 ± 1.1	6.1 ± 1.2	10.5 ± 1.7	7.5 ± 2.2
Zinc (mg/day)	6.3 ± 0.4	5.4 ± 1.5	5.4 ± 0.9	3.4 ± 1.1
Copper (mg/day)	2.2 ± 1.5	1.0 ± 0.4	1.7 ± 0.9	1.8 ± 1.2
Magnesium (mg/day)	176 ± 34	110 ± 35	248 ± 57	174 ± 70
Vitamin K1 (µg/day)	398 ± 231	176 ± 152	447 ± 410	509 ± 482
Unsaturated/saturated fat	3.5 ± 0.7	3.2 ± 0.8	3.7 ± 1.5	3.1 ± 1.0
Protein (% energy)	11.5 ± 0.6	7.6 ± 0.6	8.4 ± 0.7	3.8 ± 0.8
Fat (% energy)	32.3 ± 2.2	36.8 ± 5.0	32.6 ± 3.5	34.1 ± 4.9
Carbohydrate (% energy)	56.3 ± 2.0	55.2 ± 5.4	58.4 ± 3.8	61.3 ± 5.2
PRAL (mEq/day)	1.8 ± 9.1	−7.0 ± 6.0	−26.9 ± 15.4	−40.3 ± 13.8
NEAP (mEq/day)	42.7 ± 2.7	34.8 ± 9.8	22.2 ± 8.3	6.1 ± 3.9

Data are presented as mean ± SD for 10 menus per dietary pattern. MedRen, Mediterranean renal diet (0.8 g protein/kg); LPD, conventional low-protein diet (0.6 g/kg); sVLPD, supplemented very-low-protein diet (0.3 g/kg); PRAL, potential renal acid load; NEAP, net endogenous acid production.

**Table 4:** eADI-17 scores for the four renal dietary patterns.

Diet	Mean ± SD	Median	Interquartile Range
MedRen	11.90 ± 0.66	12.00	11.50–12.38
Vegan	9.75 ± 0.63	10.00	9.13–10.00
LPD	8.90 ± 1.10	9.00	7.75–10.00
sVLPD	8.40 ± 0.52	8.00	8.00–9.00

Data are expressed as mean ± SD, median, and interquartile range. The absolute mean difference was 3.50 points between MedRen and sVLPD and 2.15 points between MedRen and vegan, indicating descriptive separation among the selected menu sets.

**Table 5:** Pairwise differences in eADI-17 scores among the four renal dietary patterns.

Pairwise Comparison of eADI-17	*p*-Value
MedRen vs. LPD	0.000810
MedRen vs. Vegan	0.000816
MedRen vs. sVLPD	0.000810
Vegan vs. sVLPD	0.001779
LPD vs. Vegan	0.201580
LPD vs. sVLPD	0.369109

Reported values are Holm-adjusted *p*-values.

**Table 6:** Nutritional variables correlated with eADI-17 after FDR correction in pooled menu-level analyses.

Nutrient	Spearman’s ρ	FDR-Adjusted *p*-Value	Association
Total protein	0.785	0.000001	Strong positive
Phosphorus	0.727	0.000002	Strong positive
Plant protein	0.556	0.001987	Moderate positive
Zinc	0.508	0.004813	Moderate positive
Vitamin C	−0.506	0.004813	Moderate negative
Iron	0.503	0.004813	Moderate positive
Calcium	0.486	0.006506	Moderate positive
Total carbohydrates	−0.462	0.010441	Moderate negative

## Data Availability

The menu-level data supporting the findings of this study are available from the corresponding author upon reasonable request.

## References

[1] Chavda V.P., Feehan J., Apostolopoulos V. (2024). Inflammation: The cause of all diseases. Cells.

[2] Furman D., Campisi J., Verdin E., Carrera-Bastos P., Targ S., Franceschi C., Ferrucci L., Gilroy D.W., Fasano A., Miller G.W. (2019). Chronic inflammation in the etiology of disease across the life span. Nat. Med..

[3] Kadatane S.P., Satariano M., Massey M., Mongan K., Raina R. (2023). The role of inflammation in CKD. Cells.

[4] Amdur R.L., Feldman H.I., Gupta J., Yang W., Kanetsky P., Shlipak M., Rahman M., Lash J.P., Townsend R.R., Ojo A. (2016). Inflammation and progression of CKD: The CRIC Study. Clin. J. Am. Soc. Nephrol..

[5] Nilsson A., Cano A., Bergens O., Kadi F. (2021). Randomized controlled trial for promotion of healthy eating in older adults by increasing consumption of plant-based foods: Effect on inflammatory biomarkers. Nutrients.

[6] Reyneke G.L., Lambert K., Beck E.J. (2025). Food-based indexes and their association with dietary inflammation. Adv. Nutr..

[7] Shivappa N., Steck S.E., Hurley T.G., Hussey J.R., Hébert J.R. (2014). Designing and developing a literature-derived, population-based dietary inflammatory index. Public Health Nutr..

[8] Tabung F.K., Smith-Warner S.A., Chavarro J.E., Wu K., Fuchs C.S., Hu F.B., Chan A.T., Willett W.C., Giovannucci E.L. (2016). Development and validation of an empirical dietary inflammatory index. J. Nutr..

[9] Kaluza J., Hellerström L., Kaluza D., Chabok A., Åkesson A., Michaëlsson K., Wolk A. (2025). Development of empirical anti-inflammatory diet index: A cross-sectional study. Nutr. J..

[10] Cupisti A., Gallieni M., Avesani C.M., D’Alessandro C., Carrero J.J., Piccoli G.B. (2020). Medical nutritional therapy for patients with chronic kidney disease not on dialysis: The low protein diet as a medication. J. Clin. Med..

[11] Cupisti A., D’Alessandro C., Di Paolo A., Ferraro P.M., La Porta E., Noce A., Panichi V., Parisi A., Messa P. (2026). Lifestyle and environment aspects challenging kidney health. Kidney Blood Press. Res..

[12] Hu E.A., Coresh J., Anderson C.A.M., Appel L.J., Grams M.E., Crews D.C., Mills K.T., He J., Scialla J., Rahman M. (2021). Adherence to healthy dietary patterns and risk of CKD progression and all-cause mortality: Findings from the CRIC (Chronic Renal Insufficiency Cohort) Study. Am. J. Kidney Dis..

[13] Guo M., Lei Y., Liu X., Li X., Xu Y., Zheng D. (2024). Association between dietary inflammatory index and chronic kidney disease in middle-aged and elderly populations. Front. Nutr..

[14] Mazhar F., Fu E.L., Faucon A.L., Hjemdahl P., Mathisen J., Muhammad I.F., Plunde O., Perkovic V., Tuttle K.R., Carrero J.J. (2025). Systemic inflammation and the risks of adverse kidney outcomes in adults with atherosclerotic cardiovascular disease. Am. J. Kidney Dis..

[15] Stenvinkel P. (2025). Inflammation: A key driver in the progression of CKD. Am. J. Kidney Dis..

[16] Bano G., Trevisan C., Carraro S., Solmi M., Luchini C., Stubbs B., Manzato E., Sergi G., Veronese N. (2017). Inflammation and sarcopenia: A systematic review and meta-analysis. Maturitas.

[17] Pan L., Xie W., Fu X., Lu W., Jin H., Lai J., Zhang A., Yu Y., Li Y., Xiao W. (2021). Inflammation and sarcopenia: A focus on circulating inflammatory cytokines. Exp. Gerontol..

[18] Mansouri F., Jafari F., Ranjbar S., Souni F., Jahromi S.E., Shateri Z., Babajafari S., Nouri M. (2024). Dietary inflammatory index could increase the risk of sarcopenia in patients with chronic kidney disease. Sci. Rep..

[19] D’Alessandro C., Piccoli G.B., Calella P., Brunori G., Pasticci F., Egidi M.F., Capizzi I., Bellizzi V., Cupisti A. (2016). “Dietaly”: Practical issues for the nutritional management of CKD patients in Italy. BMC Nephrol..

[20] CREA Food Composition Tables. https://www.alimentinutrizione.it/tabelle-nutrizionali/.

[21] BDA Food Composition Database for Epidemiological Studies in Italy. https://bda.ieo.it/.

[22] United States Department of Agriculture FoodData Central. https://fdc.nal.usda.gov/.

[23] Remer T., Manz F. (1995). Potential renal acid load of foods and its influence on urine pH. J. Am. Diet. Assoc..

[24] Frassetto L.A., Todd K.M., Morris R.C., Sebastian A. (1998). Estimation of net endogenous noncarbonic acid production in humans from diet potassium and protein contents. Am. J. Clin. Nutr..

